# Urodynamic evaluation of neurogenic bladder in patients with spinal cord injury within 6 months post-injury: a Retrospective Cross-Sectional Study

**DOI:** 10.1038/s41393-025-01074-0

**Published:** 2025-03-24

**Authors:** Onyoo Kim, Lyekyung An, Byung Chan Lee

**Affiliations:** 1 Department of Spinal Cord Injury Rehabilitation, National Rehabilitation Center, Seoul, Korea; 2 Department of Rehabilitation Medicine, National Rehabilitation Center, Seoul, Korea; 3 Department of Physical Medicine and Rehabilitation, College of Medicine, Chung-Ang University, Seoul, Korea

**Keywords:** Neurogenic bladder, Spinal cord diseases

## Abstract

**Study Design:**

Retrospective cross-sectional survey of Korean patients with spinal cord injury (SCI) within 6 months post-injury.

**Objectives:**

To evaluate urodynamic parameters and identify unfavorable urodynamic findings in patients with neurogenic bladder due to SCI during the acute to subacute stages of the disease based on the post-injury time interval.

**Setting:**

National Rehabilitation Center, Seoul, Korea.

**Methods:**

Data from urodynamic tests performed on individuals with SCI within 6 months post-injury were collected. Based on the time interval from injury to testing, the recruited patients were divided into three groups: 0–90 days, 91–135 days, and 136–180 days. Based on these groups, urodynamic test parameters and incidence of high-risk findings (high detrusor pressure exceeding 40 cmH_2_O during the filling phase, low compliance of the bladder, and detrusor-sphincter dyssynergia [DSD]) and unfavorable urodynamic findings (detrusor overactivity [DO], underactive or acontractile bladder) were compared.

**Results:**

Analysis of urodynamic study (UDS) findings in 191 individuals with acute to subacute SCI, revealed that unfavorable urodynamic findings were observed within 3 months after injury in both complete and incomplete SCI. The UDS test results and incidence of unfavorable outcomes based on the interval between injury and examination showed no significant statistical differences over time.

**Conclusions:**

The urodynamics of individuals with SCI suggest that high-risk or unfavorable urodynamic results are common in the acute to subacute stages of SCI.

## Introduction

Neurogenic lower urinary tract dysfunction (NLUTD) is common in individuals with spinal cord injury (SCI), affecting approximately 70–84% of patients [1]. Despite considerable advancements in NLUTD management, including clean intermittent catheterization, mortality rates due to urogenital system abnormalities in individuals with SCI remain higher than in the general population [2]. Therefore, meticulous evaluation and selection of management approaches for NLUTD in individuals with SCI is crucial to protecting the upper urinary tract (UUT) and preventing long-term complications.

Urodynamic study (UDS) is the primary objective method for evaluating NLUTD and is considered the gold standard in several guidelines for NLUTD [3, 4]. Unfavorable urodynamic parameters presented in UDS are widely recognized as high-risk indicators for renal complications, and initiating proper management of NLUTD could mitigate UUT complications in individuals with SCI [5, 6]. However, to date, the timing of initial UDS and follow-up intervals have not been standardized and are typically determined by symptoms or signs reported by the patients or based on physician preferences.

The first year after SCI is particularly crucial owing to the dynamic changes in lower urinary tract functions in the patients. Therefore, early interventions in NLUTD after SCI have been recommended to prevent renal complications and modify lower urinary functions [7, 8]. Discrepancies between the symptoms and urodynamics in individuals with SCI [9] emphasizes the importance of determining the proper timing of UDS for managing NLUTD in individuals with SCI.

Therefore, describing urodynamic findings in the acute to subacute period after SCI may play a crucial role in determining the appropriate timing for UDS. This study aimed to evaluate urodynamics, detect unfavorable urodynamic findings within 6 months after SCI and compare these findings at different time intervals.

## Methods

### Participants

This was a retrospective, cross-sectional study. We conducted a retrospective chart review of individuals with SCI who had undergone UDS within 6 months of SCI at the National Rehabilitation Center, Seoul, Korea, from June 2015 to December 2022. The requirement for informed consent was waived due to the retrospective nature of the study. Inclusion criteria were as follows: (1) age > 18 years, (2) suprasacral type of SCI, (3) UDS performed within 6 months after SCI, and (4) no definite urological abnormalities before SCI (prostate hypertrophy, urological cancer, or other abnormalities that could affect UDS results). Patients with concurrent traumatic brain injury or prior neurologic disease and other traumatic damage causing urologic dysfunction (pelvic fracture, urethral injury) were excluded. We assessed the neurological status of individuals with SCI according to the ASIA/ISCoS International Standards for Neurological Classification of Spinal Cord Injury (ISNCSCI) [10]. The ISNCSCI was assessed for each individual with SCI at the time of rehabilitation admission. The included individuals were divided into two groups based on the completeness of the injury, as defined by the ISNCSCI. Both groups were further divided into three subgroups according to the time interval between SCI and UDS: within 0–90 days (3 months), 91–135 days (4.5 months), and 136–180 days (6 months). This study was approved by the local ethics committee of the National Rehabilitation Centre (NRC-2023-02-015).

### Urodynamic evaluation

All UDS procedures were conducted according to the standard methods recommended by the International Continence Society [11]. UDS was performed using a multichannel urodynamic system (Aquarius LT, Laborie, USA). Patients with suspected or confirmed urinary tract infections were deferred from evaluation until resolution of the infection. No alterations were made to the schedule or dosage of medications used to manage neurogenic bladder, such as anticholinergics or beta-agonists, before or during urodynamic evaluation. The examination was conducted with the patient in the supine position.

### Outcome measures

The outcome measures were urodynamic parameters, including the results of filling cystometry (bladder capacity, bladder compliance, and maximum detrusor pressure during the filling phase) and the results of the voiding phase (maximum detrusor pressure during the voiding phase, voided volume [VV], and post-voided residual urine [PVRU]). All parameters were based on the International Spinal Cord Injury Urodynamic Basic Data Sets formulated by the International Spinal Cord Society [12].

Cystometric bladder capacity was defined as the bladder volume at the end of the filling cystogram. Bladder compliance was calculated by dividing the change in volume by the change in detrusor pressure during filling cystometry. The maximal detrusor pressure during the filling phase was measured as the highest detrusor pressure during filling cystometry. The maximum detrusor pressure during the voiding phase was recorded. In addition, the VV and PVRU were recorded at the end of micturition.

High-risk findings (high detrusor pressure exceeding 40 cmH2O during the filling phase, low compliance of the bladder, and detrusor-sphincter dyssynergia [DSD]) and unfavorable findings (detrusor overactivity [DO], underactive or acontractile bladder) were recorded based on the observations made by the assessor during the UDS. High detrusor pressure exceeding 40 cmH_2_O during the filling phase was also recorded as an unfavorable urodynamic parameter [5]. Detrusor-sphincter dyssynergia (DSD) was defined as a detrusor contraction with an involuntary contraction of the urethral muscle recorded on electromyography [13]. Low bladder compliance was defined as a compliance value lower than 20 mL/cmH_2_O. Detrusor overactivity (DO) was defined as an involuntary detrusor contraction observed during filling cystometry. Detrusor function during the voiding phase was divided into two categories (normal, underactive, or acontractile bladder). Acontractile bladder is defined as a condition with no increase in detrusor pressure during voluntary micturition. An underactive bladder was defined as having a reduced detrusor pressure (<40 cmH_2_O) resulting in prolonged bladder emptying or failure to achieve complete emptying within a normal time span [13, 14].

### Statistical analysis

Descriptive data are presented as the mean ± standard deviation. All categorical data are presented as numbers and percentages. For basal demographic data between the complete and incomplete SCI groups, descriptive statistics were analyzed using the Student’s t-test. Pearson’s chi-square test or Fisher’s exact test was used to analyze categorical data.

To evaluate the differences between time interval subgroups, either one-way analysis of variance or the Kruskal–Wallis test was used, depending on the normality of the data, to determine between-group differences in the descriptive statistics. For categorical data within each group, the Pearson’s chi-square test or Fisher’s exact test was used. All statistical analyses were performed using IBM SPSS Statistics 23.0 (IBM Corp., Armonk, NY, USA), and a significance level of 5% was set at a 95% confidence interval.

## Results

A total of 191 individuals were recruited through a retrospective chart review. Among them, 63 individuals had motor-complete injury (American Spinal Injury Association Impairment Scale [AIS] A or B; 45 men and 18 women), and 128 individuals had motor-incomplete injury (AIS C or D; 90 men and 38 women). The average age in the complete and incomplete injury groups was 45.6 and 55.3 years, respectively. Trauma accounted for 69.8% of individuals in the complete injury group and 58.6% in the incomplete injury group. Detailed demographic data are presented in Table 1.

**Table 1:** Basal demographics of including participants.

Variables	Complete N (%) or M (SD)	Incomplete N (%) or M (SD)	Value (*p*)
Sex			0.025^a^ (*0.873*)
Male	45 (71.4)	90 (70.3)	
Female	18 (28.6)	38 (29.7)	
Age (yrs.)	45.63 (16.02)	55.30 (15.92)	−3.936^b^ (***<0.001***)
AIS			191.000^a^ (***<0.001***)
A	34 (54.0)	0 (0.0)	
B	29 (46.0)	0 (0.0)	
C	0 (0.0)	34 (26.6)	
D	0 (0.0)	94 (73.4)	
Neurological level			11.694^a^ (***0.003***)
Cervical	25 (39.7)	68 (53.1)	
Thoracic	33 (52.4)	36 (28.1)	
Lumbar	5 (7.9)	24 (18.8)	
Etiology of injury			2.274^a^ (*0.132*)
Traumatic	44 (69.8)	75 (58.6)	
Non-Traumatic	19 (30.2)	53 (41.4)	
Duration between injury and UDS (days)			1.973^a^ (*0.373*)
0–90	11 (17.5)	34 (26.6)	
90–135	33 (52.4)	61 (47.7)	
135–180	19 (30.2)	33 (25.8)	
Voiding method			35.468^a^ (***<0.001***)
Foley	22 (34.9)	25 (19.5)	
Suprapubic	1 (1.6)	2 (1.6)	
CIC	33 (52.4)	34 (26.6)	
Spontaneous Voiding	4 (6.3)	64 (50.0)	
Combined methods^c^	3 (4.8)	3 (2.3)	
Bladder medication			0.052^a^ (*0.974*)
Not used	38 (60.3)	75 (58.6)	
1	16 (25.4)	34 (26.6)	
2	9 (14.3)	19 (14.8)	

*AIS* american spinal injury association impairment scale, *UDS* urodynamic study, *CIC* clean intermittent catheterization.

^a^Pearson’s chi-squared test. ^b^Student’s t test. **Bold** is statistically significant (*p* < 0.05).

^c^Spontaneous or Crede voiding + intermittent catheterization.

Based on the time interval between UDS and SCI, the individuals were divided into three groups (0–90 days, 91–135 days, and 136–180 days). The voiding methods and detailed prescribed bladder medications for the participants are described in Table 2 and Supplementary Table 1, respectively. More than 50% of the individuals did not use bladder medication, with no statistically significant difference in the use of bladder medications according to the period, except for the use of anticholinergics in individuals with incomplete SCI (0–90 days, 2.9%; 91–135 days, 21.3%, and 136–180 days, 27.3%). (Supplementary Table 1).

**Table 2:** Voiding methods and bladder medication in participants across time-interval subgroups.

Variables	Complete			Value (*p*)	Incomplete			Value (*p*)
	0–90 N (%)	90–135 N (%)	135–180 N (%)		0–90 N (%)	90–135 N (%)	135–180 N (%)	
Voiding method				7.933^b^ (*0.380*)				6.035^b^ (*0.631*)
Foley	6 (54.5)	13 (39.4)	3 (15.8)		10 (29.4)	11 (18.0)	4 (12.1)	
Suprapubic	0 (0.0)	1 (3.0)	0 (0.0)		1 (2.9)	1 (1.6)	0 (0.0)	
CIC	5 (45.5)	16 (48.5)	12 (63.2)		8 (23.5)	17 (27.9)	9 (27.3)	
Spontaneous voiding	0 (0.0)	2 (6.1)	2 (10.5)		14 (41.2)	30 (49.2)	20 (60.6)	
Combined methods^c^	0 (0.0)	1 (3.0)	2 (10.5)		1 (2.9)	2 (3.3)	0 (0.0)	
Bladder medication				1.487^b^ (*0.846*)				7.649^a^ (*0.105*)
Not used	7 (63.6)	18 (54.5)	13 (68.4)		26 (76.5)	32 (52.5)	17 (51.5)	
1 used	2 (18.2)	10 (30.3)	4 (21.1)		7 (20.6)	17 (27.9)	10 (30.3)	
2 Used	2 (18.2)	5 (15.2)	2 (10.5)		1 (2.9)	12 (19.7)	6 (18.2)	

*CIC* clean intermittent catheterization.

^a^Pearson’s chi-squared test. ^b^Fisher’s exact test. **Bold** is statistically significant (*p* < 0.05).

^c^Spontaneous or Crede voiding + intermittent catheterization.

The continuous parameters (bladder capacity, bladder compliance, VV, PVRU, and maximal detrusor pressure during the filling and voiding phases) in both groups are summarized in Table 3. No statistically significant differences in continuous UDS parameters according to the time interval were observed between the groups.

**Table 3:** Results of urodynamics in participants across time-interval subgroups.

Variables	Date between injury and UDS			H^a^	*p*
	0–90 (N = 11)	90–135 (N = 33)	135–180 (N = 19)		
**Complete**					
Bladder capacity (mL)	546.82 (143.36)	488.03 (145.25)	561.58 (124.69)	4.623	0.099
Bladder compliance (mL/cm H_2_O)	45.86 (35.12)	69.85 (91.47)	81.77 (82.41)	1.707	0.426
VV (mL)	0.00 (0.00)	11.82 (40.27)	7.89 (34.41)	1.833	0.400
PVRU (mL)	523.64 (219.97)	470.00 (167.46)	553.16 (130.30)	4.805	0.090
Max Pdet at filling (cm H_2_O)	17.27 (14.89)	29.56 (25.05)	24.12 (20.37)	1.986	0.370
Max Pdet at voiding (cm H_2_O)	8.73 (6.97)	16.14 (16.46)	16.21 (15.74)	1.092	0.579
**Variables**	**Date between injury and UDS**			**F**^**b**^	***p***
	**0–90 (N = 34)**	**90–135 (N = 61)**	**135–180 (N = 33)**		
**Incomplete**					
Bladder capacity (mL)	503.26 (106.48)	506.48 (122.11)	506.18 (159.80)	0.007	0.993
Bladder compliance (mL/cm H_2_O)	75.28 (95.47)	91.85 (102.90)	135.75 (154.08)	2.462	0.089
VV (mL)	68.82 (135.62)	122.62 (192.64)	66.36 (137.06)	1.760	0.176
PVRU (mL)	414.12 (183.97)	369.75 (221.89)	432.42 (233.02)	1.039	0.357
Max Pdet at filling (cm H_2_O)	23.62 (20.08)	24.42 (24.21)	25.96 (28.81)	0.080	0.924
Max Pdet at voiding (cm H_2_O)	20.07 (21.65)	26.62 (25.03)	24.09 (26.92)	0.768	0.466

*UDS* urodynamic study, *VV* voided volume, *PVRU* post-voided residual urine, *Max Pdet at filling* maximal detrusor pressure at the filling phase, *Max Pdet at voiding* maximal detrusor pressure at the voiding phase.

^a^Kruskal-Wallis test.

^b^One-way ANOVA.

Table 4 summarizes the high-risk or unfavorable urodynamic findings in the complete and incomplete SCI groups. In the 0–90 days subgroup, DO was observed in 54.5% of the individuals, and a low-compliance bladder was observed in 36.4% of the individuals with complete SCI. In the 0–90 days subgroup, DO was observed in 52.9% and low-compliance bladder was observed in 20.6% of individuals with incomplete SCI. In the filling phase, a maximal detrusor pressure exceeding 40 cmH_2_O was consistently observed in both the complete and incomplete SCI groups (9.1% in the 0–90 days group, 30.3% in the 91–135 days group, and 36.8% in the 136–180 days group for the complete SCI group; 20.6% in the 0–90 days group, 18.0% in the 91–135 days group, and 21.2% in the 136–180 days group for the incomplete SCI group). However, no statistically significant difference was observed over time in either group. In the voiding phase, all individuals with complete SCI (100%) in the 0–90 days subgroup had DU or acontractile bladder, and DSD was observed in 36.4% of patients. This trend was similar in the 0–90 days subgroup of the incomplete SCI group, with a DU or acontractile bladder rate of 97.1% and a DSD rate of 35.3%. No statistically significant differences were observed in the presence of unfavorable urodynamics across all sub-groups within the complete and incomplete SCI groups (Table 4).

**Table 4:** Results of high-risk or unfavorable urodynamic findings in participants across time-interval subgroups.

Completeness	Date between injury and UDS			Value (*p*)
	0–90 (N = 11)	90–135 (N = 33)	135–180 (N = 19)	
Detrusor function at filling				
DO	6 (54.5)	20 (60.6)	9 (47.4)	0.861^a^
Normal	5 (45.5)	13 (39.42)	10 (52.6)	(*0.650*)
Bladder compliance				
Low	4 (36.4)	10 (30.3)	1 (5.3)	5.741^b^
Normal	7 (63.6)	23 (69.7)	18 (94.7)	(*0.053*)
Detrusor pressure over 40 cmH_2_O at the filling phase				
Yes	1 (9.1)	10 (30.3)	7 (36.8)	2.649^b^
No	10 (90.9)	23 (69.7)	12 (63.2)	(*0.235*)
Detrusor function at voiding				
DU or Acontractile	11 (100.0)	32 (97.0)	19 (100.0)	1.240^b^
Normal	0 (0.0)	1 (3.0)	0 (0.0)	(*1.000*)
Urethral function at voiding				
DSD	4 (36.4)	12 (36.4)	5 (26.3)	0.603^a^
Normal	7 (63.6)	21 (63.6)	14 (73.7)	(*0.740*)
**Incompleteness**	**Date between injury and UDS**			**Value (*p***)
	**0–90 (N = 34)**	**90–135 (N = 61)**	**135–180 (N = 33)**	
Detrusor function at filling				
DO	18 (52.9)	27 (44.3)	15 (45.5)	0.696^a^
Normal	16 (47.1)	34 (55.7)	18 (54.5)	(*0.706*)
Bladder compliance				
Low	7 (20.6)	12 (19.7)	8 (24.2)	0.276^a^
Normal	27 (79.4)	49 (80.3)	25 (75.8)	(*0.871*)
Detrusor pressure over 40 cmH_2_O at the filling phase				
Yes	7 (20.6)	11 (18.0)	7 (21.2)	0.171^a^
No	27 (79.4)	50 (82.0)	26 (78.8)	(*0.918*)
Detrusor function at voiding				
DU or Acontractile	33 (97.1)	54 (88.5)	27 (81.8)	4.024^b^
Normal	1 (2.9)	7 (11.5)	6 (18.2)	(*0.120*)
Urethral function at voiding				
DSD	12 (35.3)	24 (39.3)	15 (45.5)	0.733^a^
Normal	55 (64.7)	37 (60.7)	18 (54.5)	(*0.693*)

*UDS* urodynamic study, *DO* detrusor overactivity, *DU* detrusor underactivity, *DSD* detrusor-sphincter dyssynergia.

^a^Pearson’s chi-squared test.

^b^Fisher’s exact test.

## Discussion

In our study of UDS findings in 191 individuals with acute to subacute SCI, high-risk or unfavorable urodynamic findings such as DO, low bladder compliance, high maximal detrusor pressure during the filling phase, underactive bladder or DSD were common within 3 months following SCI. The UDS test results according to the time interval in individuals with SCI showed no statistically significant differences over time in patients with complete and incomplete SCIs.

NLUTD is a common complication in most individuals with SCI. Several guidelines recommend UDS as the only objective test, which should be performed at an appropriate time following SCI [3, 4]. However, the appropriate time for performing UDS remains inconclusive. In traumatic SCI, an acontractile or underactive bladder is the main finding of NLUTD during the acute period referred to as spinal shock. An acontractile or underactive bladder changes over time to other patterns of NLUTD, such as DO or reduced bladder compliance. Therefore, previous studies have suggested delaying the initial UDS for 3 months after SCI, especially in cases of complete lesions [15–17].

However, studies on early NLUTD patterns in individuals with SCI have reported inconsistent results. A study on urodynamic evaluation in individuals with SCI performed within 40 days after injury in 2018 showed that only 37% (n = 20) of patients exhibited a flaccid bladder, with DO and DSD observed in 32 (32/54, 59.3%) and 25 (25/54, 46.3%) patients, respectively, recommending early urodynamic evaluation [18]. Furthermore, a cross-sectional study involving 101 individuals with cervical SCI published in 2021 revealed statistically significant differences in urodynamic evaluation results between 0–90 days and 91–365 days post-injury between individuals with complete and incomplete SCI in aspects of prevalence of DO and DSD, suggesting that delaying the initial UDS after 3 months of SCI may be beneficial [15]. However, our findings elucidated that the probability of the occurrence of high-risk or unfavorable urodynamics, including DO, low bladder compliance, and DSD within 3 months in individuals with SCI is not uncommon, which aligns with the findings of a UDS cohort study in individuals with SCI published in 2023 [19].

Therefore, UDS should be performed at the earliest in individuals with SCI to establish a management plan for NLUTD. A significant dissociation between the symptoms experienced by patients and UDS findings has been reported, with approximately 68.75% of treatment failures identified solely based on symptoms [9]. In this retrospective chart review, 39.7% of patients who were using bladder medication before the test were included in the complete SCI group and 41.4% in the incomplete SCI group, depending on the patient’s symptoms. Nevertheless, considering the proportion of high-risk or unfavorable UDS findings during UDS evaluation, an early UDS can facilitate NLUTD management in individuals with SCI.

Second, the timing of follow-up for UDS should be considered. Currently, there are no clear guidelines on when to schedule follow-up UDS for individuals with SCI. The consensus among clinicians is to perform follow-up UDS when new complications arise or when signs or symptoms change [3, 4]. However, based on time intervals, our findings revealed that statistically significant differences in UDS parameters were not observed in individuals with SCI within 6 months after injury. Furthermore, considering the difficulties, costs, and potential complications of UDS testing [20–22], it may be reasonable to consider scheduling follow-up testing at least 6 months after the injury, excluding patients with a high risk of UUT involvement after the initial evaluation.

However, this study had several limitations. First, this was a retrospective chart review; therefore, repeated testing in patients could not be performed, which limits the ability to observe changes in urodynamics within individuals. Second, UDS was not performed after discontinuation of bladder medication; therefore, the potential influence of bladder medication on the urodynamic results could not be determined. Moreover, anticholinergics or mirabegron can alter the maximal cystometric capacity, detrusor pressure, and bladder compliance in neurogenic bladder [23, 24]. Therefore, these factors must be considered. Third, this study included a smaller sample size. Future prospective analyses involving long-term and repeated examinations are necessary to address these limitations.

Urodynamics in the acute to subacute stages of SCI suggested that high-risk or unfavorable urodynamic results (DO, high detrusor pressure, reduced compliance, or DSD) were common within 6 months of SCI. Therefore, initial UDS should be performed as early as possible for the management of NLUTD in individuals with SCI. Moreover, no significant differences were observed between the UDS results in the time-interval subgroups of participants within 6 months of the injury, indicating that the follow-up UDS schedule could be delayed by at least 6 months after injury in patients with a low risk of UUT involvement.

## Supplementary information

Supplemental Table

STROBE Statement

## Data Availability

The datasets generated during and/or analyzed during the current study are available from the corresponding author on reasonable request.
