# Supplementary material for: Urodynamic evaluation of neurogenic bladder in patients with spinal cord injury within 6 months post-injury: a Retrospective Cross-Sectional Study
Source: Spinal Cord. 2025 Mar 24;63(5):246–51. doi: 10.1038/s41393-025-01074-0 (PMC12074986; doi:10.1038/s41393-025-01074-0)
Supplement: Supplementary file 1 — Supplemental Table [file 41393_2025_1074_MOESM1_ESM.docx]

**Supplemental Table 1.** Detailed bladder medications in participants across Time-interval Subgroups**.**

| Variables | Complete | | | Value  (*p*) | Incomplete | | | Value  (*p*) |
| --- | --- | --- | --- | --- | --- | --- | --- | --- |
|  | 0-90  N (%) | 90-135  N (%) | 135-180  N (%) |  | 0-90  N (%) | 90-135  N (%) | 135-180  N (%) |  |
| Anticholinergics |  |  |  |  |  |  |  |  |
| Not used | 8  (72.7) | 24  (72.7) | 14  (73.7) | 0.006^a^ | 33  (97.1) | 48  (78.7) | 24  (72.7) | 7.609^a^ |
| Used | 3  (27.3) | 9  (27.3) | 5  (26.3) | (*.997*) | 1  (2.9) | 13  (21.3) | 9  (27.3) | (***.022***) |
| Beta agonists |  |  |  |  |  |  |  |  |
| Not used | 9  (81.8) | 24  (72.7) | 17  (89.5) | 1.972^b^ | 33  (97.1) | 52  (85.2) | 30  (90.9) | 3.204^b^ |
| Used | 2  (18.2) | 9  (27.3) | 2  (10.5) | (*.413*) | 1  (2.9) | 9  (14.8) | 3  (9.1) | (*.165*) |
| Alpha blocker |  |  |  |  |  |  |  |  |
| Not used | 10  (90.9) | 32  (97.0) | 18  (94.7) | 1.316^b^ | 29  (85.3) | 46  (75.4) | 26  (78.8) | 1.282^a^ |
| Used | 1  (9.1) | 1  (3.0) | 1  (5.3) | (*.747*) | 5  (14.7) | 15  (24.6) | 7  (21.2) | (*.527*) |
| Cholinomimetics |  |  |  |  |  |  |  |  |
| Not used | 11  (100.0) | 32  (97.0) | 19  (100.0) | 1.240^b^ | 32  (94.1) | 57  (93.4) | 30  (90.9) | 0.457^b^ |
| Used | 0  (0.0) | 1  (3.0) | 0  (0.0) | (*1.000*) | 2  (5.9) | 4  (6.6) | 3  (9.1) | (*.821*) |

**Bold** is statistically significant(*p*<.05).

^a^: Pearson’s chi-squared test; ^b^: Fisher’s exact test.
